# Understanding the global dynamics of continuing unmet need for family planning and unintended pregnancy

**DOI:** 10.1007/s42379-023-00130-7

**Published:** 2023-04-05

**Authors:** Justine Coulson, Vinit Sharma, Hua Wen

**Affiliations:** 1 United Nations Population Fund, China Office, Beijing, China; 2 United Nations Population Fund, Asia Pacific Regional Office, Bangkok, Thailand

## Current status of unmet need for family planning 

Family planning is regarded as one of the major public health successes in the past 70 years. Worldwide, the contraceptive prevalence rate (CPR) for women of reproductive age rose from 28% in 1970 to 48% in 2019 and demand satisfied rose from 55 to 79% in the same time period (Haakenstad et al., 2022). Family planning offers both health and social benefits for women. It saves lives by preventing unintended, unwanted and unplanned pregnancies thereby reducing the need for abortions (that can often be unsafe and illegal) and also by reducing the probability of a woman’s death because of causes related to pregnancy and childbirth. In 2022, use of contraception averted more than 141 million unintended pregnancies, 29 million unsafe abortions, and almost 150,000 maternal deaths (United Nations Population Fund [UNFPA], 2022a). A number of research studies have documented that women who have planned and adequately spaced pregnancies give birth to healthier children and evidence also shows that expanding contraceptive use can lead to improvements in women’s agency and labour force participation.

Universal access to family planning is a human right, central to gender equality and women’s empowerment, and a key factor in reducing poverty and achieving the goal of Universal Health Coverage (UHC) (Prata et al., 2017). It is a very cost-effective public health intervention because of the high returns that it yields. Every US$1 invested in meeting the unmet need for contraceptives can yield up to US$120 in accrued annual benefits in the long-term; US$ 30–50 in benefits from reduced infant and maternal mortality and US$ 60–100 in long-term benefits from economic growth (FP2020, 2018).

Unmet need refers to women of reproductive age who wish to avoid a pregnancy but are not using a contraceptive method. Despite the multiple benefits of family planning and improvements in access, in 2019, an estimated 160 million women and adolescents globally had an unmet need for family planning with over half of the women with unmet need living in Sub-Saharan Africa and South Asia (Haakenstad et al., 2022). High unmet need leads to high rates of unintended pregnancies and the links between unmet need for family planning, unintended pregnancies, and unsafe abortions leading to maternal deaths, is well established. Terminations of pregnancies indicate a high unmet need for contraception, and unintended pregnancies have been identified as the underlying cause for nearly all abortions, many of which are performed illegally and under unsafe conditions.

Adolescent girls are a group that globally have substantial unmet need for contraception that results in adverse health effects and negative consequences for their development. In low- and middle-income countries (LMICs), unmet need for modern contraception is disproportionately higher among adolescent girls aged 15–19 (43%) than amongst all women aged 15–49 (24%). Adolescents in LMICs have an estimated 21 million pregnancies each year, of which approximately 50% are unintended, and 55% of unintended pregnancies end in abortions, which are often unsafe (Sully et al., 2020). Consequences of unintended pregnancies for adolescent girls can be far-reaching, including school dropout, poor sexual and reproductive health, cultural stigmas and social pressures, as well as lost opportunities for employment and income in the long term.

Whilst this Special Issue uses the standard definition of unmet need, in recent years there has been increasing debate amongst academicians and practitioners regarding the limitations of the term. For example, unmet need does not measure whether or not a woman wants to use a contraceptive method, yet half of the women classified as having an unmet need report that they do not want or intend to use a contraceptive method in the future. Should non-contraceptive use amongst these women be classified in the same way as those women who do express a desire to use a contraceptive method? (Fabic, 2022). There is also a concern that the measurement of unmet need refers to ‘sexually active women’ which suggest frequent, regular sexual activity which does not adequately capture the needs of unmarried adolescent girls in temporary or short-term unions. Furthermore, some researchers are now questioning the definition because it assumes that all women who are using a method have had their specific needs met. However, the current definition undercounts the number of women with a true unmet need for contraception as it misses the many women who are using a method that does not meet their preferences. (Rominski & Stephenson, 2019).

## Barriers to service uptake that contribute to unmet need

To address unmet need for family planning, barriers to uptake need to be approached from three perspectives: the supply, the provider and the consumer.

### Availability of quality commodities

Over time, a lot of research has gone into ensuring good manufacturing practices and setting international standards and specifications for the quality of different methods, thereby ensuring that contraceptive supplies are of high quality. New methods have been developed, research is continuously being promoted and the efficacy of available methods is being improved to reduce failure rates. Similarly, governments and donors have invested heavily in strengthening national supply chain systems, leading to improved forecasting, procurement, distribution, data and stock handling which in turn has helped to reduce stock outs and ensure access to quality commodities (PRB, 2013; CDC 1999).

When we consider the link between commodity availability and unmet need, it is important to recognize that it is not just a matter of any contraceptive method being available; the consistent availability of a range of methods is important. For example, in Asia Pacific, there is skewed method mix in 60% of the countries in the region (i.e. a single method accounts for 45% or more of the method mix) and there is often a disconnect between the reason for demand and the method used (United Nations Population Division, 2015). Research has shown that in some contexts a balanced method mix combined with a greater number of facilities can lead to a higher contraceptive uptake (Mallick et al., 2020). However, a study across 5 countries in Africa and Asia found that stock outs in service delivery points were highly unpredictable: whilst at least one method was in stock at any one time, there were huge variations in frequency of stock outs by method and type of service delivery point making it difficult for women to know where and when to access their FP service (Muhoza et al., 2021). Other studies have found that the public sector tends to have higher stocks out of long-acting reversible contraceptives (LARCs) than short term methods (Githinji et al., 2022) and displaced adolescent girls and women can be especially badly affected as when IDP camp clinics have stock outs or close, the cost of accessing services in the public sector outside the camp can be prohibitively high (Kagestan et al., 2017).

### Quality of care

It has long been recognized that high quality, client-centred care can increase the use of family planning, just as poor quality of care can contribute to unmet need (Creel et al., 2002). Provider bias undermines quality of care as providers impose unnecessary barriers to choice based on characteristics of a client, such as age, marital status and lifestyle, or unfounded beliefs about a method (Solo & Festin, 2019). The updated FP Quality of Care framework (Jain & Hardee, 2018) identifies 6 critical elements for quality FP counselling including the availability of a trained and competent provider, a two-way exchange of information between provider and client and confidential, respectful interpersonal relations. Among sexually active, never-married women, 19% cite concerns about side effects as their reason for not using a method and 49% cite infrequent sex (Sedgh & Hussain, 2014). Therefore, quality counselling that ensures all sexually active women understand the risk of becoming pregnant and the range of methods available to meet their needs remains key.

Discontinuation of contraception contributes to unmet need. In 2015, globally, 38% of women with unmet need had used a modern contraceptive method in the past but had discontinued it, and across LMICs, up to 27% of women cited reasons for discontinuation related to the service environment, including service quality and availability of sufficient choice of methods (Castle & Askew, 2015).

As well as the quality of family planning counselling, evidence shows that the routine integration of quality FP counselling into the delivery of other health services, such as ANC/PNC visits and HIV testing, can lead to an increased uptake of family planning, and therefore a reduction in unmet need (Amour et al., 2021; Dev et al., 2019). However, integration needs to be done in such a way as to ensure the quality of the family planning counselling is maintained. Where there are long waiting times, a short time with the provider and a lack of comprehensive information about FP choices, client dissatisfaction can be high and lead to the non-uptake of a method and therefore, greater unmet need (Puri et al., 2020).

### Lack of knowledge and awareness

Women’s lack of knowledge about contraception as a reason for non-use declined substantially between the 1980s and the 2000s and now, in most countries, only 0–4% of married women with unmet need demonstrate a complete lack of knowledge of family planning (Hussain et al., 2016). However, it is important to recognize that there continue to be particular groups of women with lower levels of knowledge of family planning and of particular methods and that this can contribute to higher levels of unmet need among these groups.

For adolescent girls, unplanned pregnancy and STIs continue to be a major concern globally and lack of knowledge and awareness contribute to their unmet need for contraception (Vázquez-Rodríguez et al., 2018). Global analysis suggests that CPR at first intercourse is under 60% (Wang et al., 2020). With reference to knowledge gaps about specific methods, in some contexts, adolescent girls do not consider themselves eligible for LARCs as young, unmarried women and unlike the data for adult women, higher levels of education are not associated with higher awareness (Kirubarajan et al., 2022). Furthermore, vulnerable sub-groups of adolescent girls are more likely to lack adequate knowledge of contraception contributing to higher levels of unmet need. For example, for displaced and refugee adolescent girls living in conflict- and disaster-affected populations, knowledge of a full range of contraceptives is limited and lower than that of adult women in the same population leading to unmet need and unintended pregnancies (Ivanova et al., 2018). Increasing access by adolescents to comprehensive sexuality education (CSE) could help to reduce rates of unintended pregnancy and unmet need amongst this population (UNESCO, 2015).

For post-partum women in low- and middle-income countries, uptake of contraception after pregnancy can be low due to an underestimation of the risk of pregnancy and a lack of knowledge of the possible methods that can be used (Dev et al., 2019). Unmet need for family planning has found to be higher amongst women living with HIV and is linked to a lack of access to FP information tailored to their needs (Meckie et al., 2021).

## The importance of sustainable financing

Family planning is a critical investment for the achievement of the SDGs, an essential health service and a basic human right that requires consistent funding year-on-year, regardless of national and global events. Yet, as the size of national populations increase, the cost of addressing unmet need and increasing contraceptive coverage will rise.

One of the main challenges in accelerating the reduction of the unmet need for family planning is the increase in the number of women of reproductive age globally, which rose from 1.3 billion in 1990 to 1.9 billion in 2021, an increase of 46 per cent. There was an even larger increase in the number of women of reproductive age who have a need for family planning. More than 1 billion women of reproductive age (15–49) live in low- and lower-middle-income countries. An estimated 371 million of those women are now using a modern method of family planning, 87 million more than just a decade ago. Moreover, women are demanding and using modern contraception in ever greater numbers, in every region, despite every obstacle. Even in the face of COVID-19, which caused enormous disruptions to health systems, the demand for modern contraception has continued to grow (FP2030, 2022).

UNFPA (2019) estimates that it will cost $68.5bn to end unmet need for family planning by 2030. Whilst some national governments have committed to increase domestic financing for family planning, donor funding and out-of-pocket (OOP) expenditure continues to account for 69% of expenditure on family planning in low- and middle- income countries in 2019 (FP2030, 2021). It is estimated that in 2017 OOP expenditure on contraceptive commodities in low- and middle-income countries totaled $2.09 billion (HIPs, 2018) and the need for OOP expenditure means that the poorest can experience a financial barrier to family planning access that leads to unmet need (Miller et al., 2018).

Following the London Summit on Family Planning in 2012, there has been an overall trend of rising donor funding with the 2021 total approximately $200 m higher than in 2012. However, recent years have been more challenging. Bilateral donor funding for family planning in 2020 totaled US$1.40 billion, a drop of more than US$100 million from 2019 and remained at more or less the same level in 2021 ($1.41 m) (Wexler et al., 2022) and is projected to decline further over the coming decade.

The situation is challenging for middle-income countries (MICs) as major donors are shifting funding for a range of health programmes from MICs to the poorest countries with the highest burdens of disease. This transition in family planning donor funding is especially challenging for those lower-middle-income countries where donor funding is declining at a time when demand for contraception is still increasing (Pharos Global Health Advisers, 2019).

With the decline in donor funds, domestic resource mobilization has become increasingly important element of sustainable financing for family planning. 44 of the 48 FP2020 commitment-making countries included a domestic financing commitment and domestic public financing is recognized as a High Impact Practice for accelerating FP uptake (HIPs, 2018). However, a public commitment to use domestic financing is only the first step and not all commitments have translated into actual expenditure. Budget allocation needs to be sufficient to cover all components of a national family planning programme, including commodities, service delivery and demand generation and to meet the needs of the entire population (Wexler et al., 2022). The budget needs to be fully executed -WHO (2016) estimates that between 10 and 30% of approved health budgets in Africa are unspent—and spent efficiently.

With the political declaration at the 74th session of the United Nations General Assembly in 2019 committing world leaders to achieve universal health coverage by 2030, the integration of family planning into national UHC strategies as part of a broader SRHR package of services has become a key approach to achieving more sustainable domestic financing, and it can promote a higher mCPR and lower unmet need by reducing financial barriers to access (Ross et el., 2018).

However, whether family planning is integrated into the national health benefits package or funded as a vertical programme, the potential for adequate domestic financing can be limited by the conflict with other health and non-health priorities and family planning advocates need to demonstrate the cost-effectiveness of investing in family planning.

During humanitarian crises, with shifting priorities, government funding is often diverted to life-saving efforts (rescue, relief and rehabilitation) and sexual and reproductive health services do not receive the attention they deserve (Hall et al., 2020; Noor et al., 2022). Furthermore, even within the health care facilities, the immediate focus is on life-saving interventions and on treating cases of the infection, meaning that often family planning services are neglected. This was documented during the Ebola epidemic in West Africa (Elston et al., 2017; Jones et al., 2016; Parpia et al., 2016). In a modelling exercise undertaken by UNFPA, it was visualized that during the early phases of the COVID-19 pandemic, the combined effects of various programmatic factors and challenges on the availability of contraceptive services could result in a dramatic spike in the unmet need for family planning. In the worst-case scenario, it was projected that 32% of women of reproductive age could be unable to meet their family planning needs in 2020, with the effects of such disruption in access to services continuing to have an impact until the end of the decade (Sharma et al., 2020; UNFPA, 2021a).

## Gender inequality and unmet meed for family planning

Gender inequality is a key driver of unintended pregnancies. Women are more likely to experience an unintended pregnancy when they have fewer choices and less power. The State of World Population report 2022 reveals that gender inequality is the strongest of all correlations of unintended pregnancy. Countries (and territories) with higher levels of gender inequality, as measured by the gender inequality index (GII), had higher rates of unintended pregnancy in 2015–2019 in both low- to middle-income countries and high-income countries (UNFPA, 2022b). Many factors, rooted in gender inequality, are stripping women of their fundamental decision-making power over their bodies.

The ability to decide whether or not to become pregnant is fundamental to bodily autonomy, yet, globally, where data are available, only half of women aged 15 to 49 make their own decisions regarding sexual and reproductive health and rights, about a quarter of women are unable to say no to sex, and nearly 10% are unable to make their own decisions about contraception (United Nations, 2022). Too often women are not able to exercise their autonomy on these issues due to harmful social and gender norms. Rigid gender norms and patriarchal structures that give an advantage to men over women leave women with less power in negotiating sex, contraception, and pregnancy with their husbands and partners. While contraceptive use is increasing throughout the world, opposition from others, such as husbands or family members continues to be a significant reason for women not using contraception they need (UNFPA, 2022b).

When gender inequality intersects with other forms of structural and systemic discrimination and marginalization, it increases vulnerabilities related to unintended pregnancy. Some marginalized groups, for example, sex workers and women with disabilities, often face more risk of sexual violence and legal and social barriers to contraceptive use, leading to high numbers of unintended pregnancies (Ampt et al., 2018; Faini et al., 2020; Horner-Johnson et al., 2020; UNFPA, 2018). Inequalities in sexual and reproductive health and rights also correlate with economic inequality. The lack of financial independence of women limits their autonomy in choice and makes it more difficult for them to afford contraceptive methods of choice. Within most developing countries, the unmet demand for family planning is generally greatest among women in the poorest 20% of households. Without access to contraception, poor women, particularly those who are less educated and live in rural areas, are at heightened risk of unintended pregnancy (UNFPA, 2017).

Gender-based violence driven by gender-unequal power is often associated with an increase in unintended pregnancy. Results from the WHO Multi-country study reveals that intimate partner violence (IPV) is a consistent and strong risk factor for unintended pregnancy and abortion across a variety of settings. Reducing IPV by 50% could potentially reduce unintended pregnancy by 2–18% and abortion by 4.5–40%, according to population-attributable risk estimates (Pallitto et al., 2013). In some studies, women experiencing IPV are twice as likely to have a male partner refuse to use contraception and twice as likely to report an unintended pregnancy compared to women who have not experienced violence (Silverman & Raj, 2014). The risk of sexual violence and unintended pregnancies for women and girls is exacerbated in humanitarian crises and fragile settings due to the breakdown of normal protection structures and support, and the disruption of access to contraceptives. One review of sexual violence among refugees and internally displaced persons in 19 countries estimated the prevalence of sexual violence to be 21.4% based on the 19 selected studies (Vu et al., 2014).

## Overview of papers in the special issue

This special issue of China Population and Development Studies presents original research in unmet need for contraception and unintended pregnancy including one paper from East and Southern Africa, one paper from Bangladesh, and three papers from China.

In line with global research that has shown that particular groups of adolescent girls and women face specific barriers to accessing services and therefore higher unmet need, **INNOCENT MODISAOTSILE et al.** present research that shows the intersecting challenges sex workers across 14 countries in East and Southern Africa faced in accessing services during stringent COVID-19 containment measures. Whilst many women struggled to access sexual and reproductive health services at this time, the research highlights how the stigma that sex workers faced made it especially challenging for them to access services and therefore, putting them at risk of unintended pregnancy. Rules limiting mobility outside the home negatively affected the income of sex workers, whilst the risk of strict enforcement of those rules combined with bias against sex workers made them particularly vulnerable to violence by the police. The stigma sex workers face was heightened during COVID-19 as they were labelled as vectors of the coronavirus. As a result, they were often shunned by health service providers and many sex workers were unable to access essential services such as HIV treatment, contraceptive counselling and safe abortion services. The findings of the paper can be used to inform future pandemic responses to ensure they are more inclusive of the specific needs of sex workers.

Using data generated from the China Fertility Survey 2017, **HUI WANG et al.** analyze unintended pregnancy and influencing factors among married women aged 15–49 in China. The analysis found that the incidence of unintended pregnancies among married women in China was 42.4‰ in 2017. Of women of childbearing age who had a history of pregnancy from 2010 to 2017, 22.9% of their pregnancies were unintended. Unintended pregnancies have a great impact on the number of induced abortions. Between 2010 and 2017, of all unintended pregnancies, 71.9% ended in induced abortion, and only 19.9% ended with a live birth. The incidence of unintended pregnancy and induced abortion to terminate unintended pregnancy were higher among women who live in an urban rather than a rural area, who previously given birth to a boy, who has a large number of children, who are of relatively older childbearing age, or who have a shorter inter-pregnancy interval. The analysis shows that for post-abortion contraception, only 37.3% of women chose long-term contraceptive methods after an induced abortion caused by an unintended pregnancy. The research finds that China’s fertility policy adjustment in 2016 had no significant impact on the incidence of unintended pregnancy. This contradicts the prior assumption that the loosening of the fertility policy would stimulate to a certain extent people's desire to have children.

Using data from the 2017 China Fertility Survey, **YONGAI JIN and WEIBO HU** analyze associations between women’s economic opportunities and induced abortion to test the “diverging destinies” theory which states that women with the most economic opportunities often obtain gains while women with the least economic opportunities suffer from losses. Whilst between 1985 and 2014, women with more economic opportunities were more likely to have an abortion, when the two-child policy was introduced, this trend reversed and women with good economic opportunities become less likely to have an abortion than women with poor economic opportunities. Unintended pregnancies place greater pressure on economically disadvantaged, younger and unmarried women and therefore they are more likely to seek an abortion.

Post-partum family planning (PPFP) supports healthy spacing and prevents unintended pregnancy and yet, globally, many women do not take a family planning method after giving birth, often because they underestimate the risk of pregnancy. **YUYAN LI et al**.’s study of postpartum contraceptive use in China similarly finds that over a third of women are not using a family planning 6 months after giving birth and a much higher percentage of women who do not use a PPFP method experience a pregnancy within 24 months of giving birth in comparison to those whose contraceptive demands are satisfied. Interestingly, women who had had an abortion prior to giving birth were more likely to not use a modern method of PPFP. The paper calls for the integration of PPFP into the continuum of pre-natal, obstetric and post-natal care services so that all post-partum women receive sufficient counselling on the risks of unintended pregnancy and the benefits of family planning.

Using data from survey reports such as Bangladesh Demographic and Health Survey (BDHS) 2017–2018, Multiple Indicator Cluster Survey (MICS) 2019, and Bangladesh Adolescent Health and Wellbeing Survey (BAHWS) 2019–2020, and through analyzing of relevant research papers, survey reports, and policy document, **MOHAMMAD MAINUL ISLAM and MAYABEE ARANNYA** found that although Bangladesh has policies designed to support youth rights and access to comprehensive sexuality education and relevant services, there are immense implementation gaps. Social stigma and taboos are overpowering the implementation of policies that need critical attention. Also, interventions are needed to address the significant gap in data on unmarried adolescents and their use of family planning services, which limits the analysis of the current situation of unmarried adolescents.

The five papers included in this special issue present data and analysis of unmet need for contraception and unintended pregnancy in East and Southern Africa, Bangladesh and China. These findings show that unmet need for contraception and unintended pregnancy remain important public health concerns in different geographic settings, and there are strong associations of unmet need for contraception and unintended pregnancy with demographic, socio-economic, and cultural context variables, as observed in numerous studies. Influencing factors such as age, social position, marriage status, children ever born, economic status exert significant influences over unmet need for contraception and unintended pregnancy. Findings also reaffirm that social stigma other than physical access to services prevents women from using family planning, especially for some marginalized groups such as sex workers and unmarried adolescents and women. There is a need for further study and analysis of barriers including social and cultural factors of unmet need for contraception in different social contexts to inform policies and programs that remove obstacles and promote women and adolescent girls to enjoy their bodily autonomy.
